# Improving Early Detection of Dementia in Low‐Resource Languages: A Connected Speech Analysis Case Study in Amis Language

**DOI:** 10.1002/alz.095345

**Published:** 2025-01-09

**Authors:** Chingching Lu, Shih‐Wei Chen

**Affiliations:** ^1^ National Tsing Hua University, Hsinchu City, Taiwan Taiwan

## Abstract

**Background:**

Early detection of dementia is critical for effective management and possible mitigation of the disease’s progression. Connected speech analysis offers a promising approach to detect early cognitive impairments by evaluating subtle changes in spontaneous language production. This is particularly challenging in low‐resource languages, where linguistic barriers prevent effective communication and analysis in clinical settings.

**Method:**

We utilized the BERT model, a useful machine learning algorithm, for text classification in a supervised learning setting. Initially, our dataset comprised 80 scripts in Amis, a language with limited resources spoken in rural areas of Eastern Taiwan. To augment our dataset and improve model robustness, we translated English descriptions from picture‐based narrative tasks of dementia patients and cognitively normal individuals into Amis, incorporating these into the training process.

**Result:**

After incorporating the translated datasets and retraining, the model’s ability to classify dementia and non‐dementia cases from spontaneous speech in Amis improved, reaching an overall predictive accuracy of over 80%. This suggests that data augmentation with translated content can significantly enhance model performance in low‐resource language settings.

**Conclusion:**

Our findings underscore the potential of using advanced text classification techniques to facilitate early dementia detection in underrepresented linguistic communities. This approach can be potentially replicated across other low‐resource languages, contributing to global health initiatives in dementia care. It highlights the advancements in utilizing machine learning for health interventions in linguistically diverse and resource‐limited populations.

